# Theoretical and Experimental Studies of the Shock-Compressed Gas Parameters in the Welding Gap

**DOI:** 10.3390/ma17010265

**Published:** 2024-01-04

**Authors:** Andrey Malakhov, Igor Denisov, Nemat Niyozbekov, Ivan Saikov, Denis Shakhray, Vasily Sosikov, Andrey Emelyanov

**Affiliations:** 1 Merzhanov Institute of Structural Macrokinetics and Materials Science (ISMAN), Russian Academy of Sciences, 142432 Chernogolovka, Russia; malakhov@ism.ac.ru (A.M.); denisov@ism.ac.ru (I.D.); revan.84@mail.ru (I.S.); 2 Federal Research Center of Problems of Chemical Physics and Medicinal Chemistry of Russian Academy of Sciences, 142432 Chernogolovka, Russia; shakhray@icp.ac.ru (D.S.); vaso@icp.ac.ru (V.S.); emelyanov@ficp.ac.ru (A.E.)

**Keywords:** explosive welding, shock-compressed gas, detonation velocity, high-speed photography, optical pyrometry

## Abstract

This work is devoted to the study of the processes that take place in the welding gap during explosive welding (EW). In the welding gap, when plates collide, a shock-compressed gas (SCG) region is formed, which moves at supersonic speed and has a high temperature that can affect the quality of the weld joint. Therefore, this work focuses on a detailed study of the parameters of the SCG. A complex method of determining the SCG parameters included: determination of the detonation velocity using electrical contact probes, ceramic probes, and an oscilloscope; calculation of the SCG parameters; high-speed photography of the SCG region; measurement of the SCG temperature using optical pyrometry. As a result, it was found that the head front of the SCG region moved ahead of the collision point at a velocity of 3000 ± 100 m/s, while the collision point moved with a velocity of 2500 m/s. The calculation of the SCG temperature showed that the gas was heated up to 2832 K by the shock compression, while the measured temperature was in the range of 4100–4400 K. This is presumably due to the fact that small metal particles that broke off from the welded surfaces transferred their heat to the SCG region. Thus, the results of this study can be used to optimize the EW parameters and improve the weld joint quality.

## 1. Introduction

Explosive welding (EW) is a solid phase welding process in which the detonation of explosives is used to obtain a welding joint [1]. One of the advantages of EW is that it can produce a welding joint of the materials that are difficult or impossible to weld using conventional fusion welding methods [2,3]. For example, it can be used to join dissimilar metals, or metals with big difference in their melting points [4,5,6,7]. However, EW has a number of disadvantages too. For instance, it can be difficult to control the EW parameters, which can worsen the quality of the welding joint. Nevertheless, EW has applications in various industries, such as aerospace, automotive, and defense industries [8,9,10,11,12].

The explosive nature of this welding technique, however, poses a number of challenges. For one, the shock waves generated by the detonation of the explosives make it difficult to directly monitor the welding process. On the other hand, the high pressure generates such a high mechanical stress in the metals that it is almost impossible to analyze their properties during the welding process. As a result, researchers have to make certain assumptions when studying the mechanisms of weld joint formation.

The welding joint is formed by the high velocity collision between the flyer plate and the base plate [13]. As a result, the collision point forms, which then moves in the same direction as the explosive welding process. The velocity of this collision point depends on the type of the plate geometry. There are parallel and inclined plate geometries. In the case of the parallel plate geometry, the collision point velocity (ϑ_c_) is equal to the detonation velocity (D).

**Figure 1:**
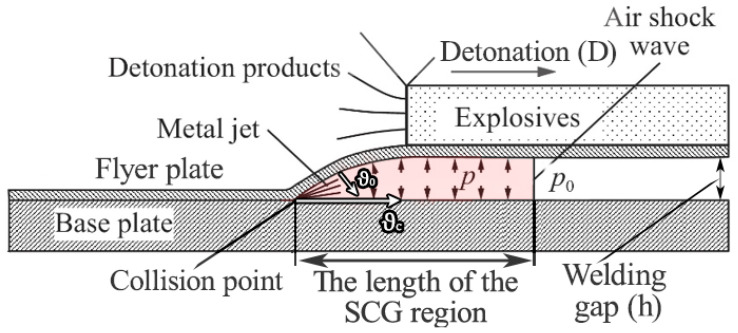
The schematic diagram of EW process [15].

It is well known that strength of the EW joint depends on the following parameters: detonation velocity (D), flyer plate velocity (ϑ_0_), welding gap (h), explosive mass ratio (r), surface finish of the initial materials, and the type of atmosphere in the welding gap (air or an inert gas) [14]. The collision of the plates forms a shock-compressed gas (SCG) and metal jet in the gap between the plates [15,16,17]. The SCG is limited on one side by the air shock wave front and on the other by the inclined surface of the flyer plate moving at collision point velocity [18]. The length of the SCG region (Figure 1) affects the quality of the weld joint and it increases in the EW process [19]. This increase is especially substantial in plates with widths of ≥0.5 m, because larger widths impede the outflow of SCG from the welding gap sideways [17]. This lengthening of the SCG region raises the temperature in the welding gap up to 3000 K [19], which leads to the heating of the welded surfaces and their local melting before the collision of the plates takes place [20]. All this must be taken into account when calculating the EW parameters, which also depend on the SCG parameters, such as velocity, pressure, and temperature.

SCG and the metal jet are usually studied through optical, radiographic (X-ray), photoelectric, and thermocouple methods. For example, in [21,22] an optical method showed a metal jet formation before the collision point. Similar results were obtained in [23], where a radiographic method was used. The important thing to note is that SCG in the welding gap is poorly seen in the X-ray images due to the low density of SCG. Furthermore, the formation of the metal jet before the collision point was simulated by the smoothed-particle hydrodynamics (SPH) method [24,25,26,27,28], which showed that the temperature of the metal jet may reach 3000 K [21]. The SPH method is a computational method used for simulating the mechanics of the continuum media, such as solid mechanics and fluid flows. But this method cannot allow for all conditions under which the EW occurs.

The authors in the study [19] mentioned the use of the photoelectric method of determining the temperature of SCG. This method yielded an exact value of the temperature of SCG, and the effects of this temperature on the surfaces were evaluated.

In [29], the authors found that SCG heats the surfaces of the plates up to 1000 °C before the collision occurs. This was shown by the thermocouple method. The heating may result in a worsening of the mechanical properties of the welding joint due to an intensified interaction between the chemical elements in the air and those on the surfaces. This interaction forms brittle oxides and nitrides in the weld interface which creates defects in the weld joint [30].

Notably, the thermocouple and photoelectric methods are based on the measurements of the voltage. Therefore, these methods are an indirect way of yielding the temperature values. Also, these methods employ highly sensitive and precise equipment that can be quite expensive and difficult to service. In addition, the thermocouple method is not adapted because of the notion of time constant, which is definitely too long with respect to the duration of EW. Therefore, this method is not suitable in all situations.

The primary goal of this study is to conduct careful experiments to determine the SCG parameters during the EW of AlMg6 alloy with 12Cr18Ni10Ti steel. The EW of AlMg6 alloy to 12Cr18Ni10Ti steel is a pressing issue in materials science at the moment. During EW, melting phases with high fusibility (Al, Mg, etc.) could also precipitate from alloys. They reduce the strength properties of the bimetal and have a negative influence on the weld joint. The EW of AlMg6 to 12Cr18Ni10Ti steel is understudied. The optimal parameters for this type of welding should be obtained.

To attain the goal, optical pyrometry and high-speed photography methods were used. Also, the temperature of SCG in the welding gap was calculated. In addition, the detonation velocity was determined using electrical contact/ceramic probes and a digital oscilloscope. The results of this study can be used to optimize EW parameters and thus improve the welding joint quality.

## 2. Materials and Methods

**Figure 2:**
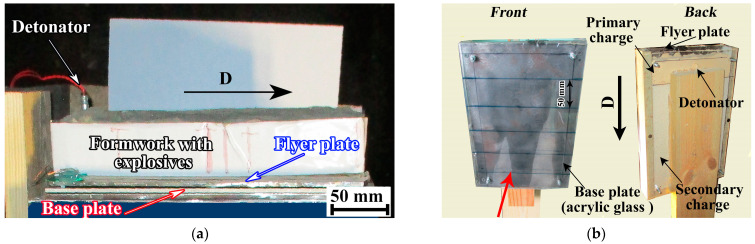
Photographs of configurations: (**a**) configuration 1; (**b**) configuration 2.

For this work, a complex method for determining the SCG parameters in the welding gap was developed. The detonation velocity was determined using electrical contact/ceramic probes and a digital oscilloscope. Then, the thermodynamic parameters of SCG were calculated. To confirm this calculation experimentally, two configurations were developed to detect the motion of SCG in the welding gap. Another scheme was developed to measure the SCG temperature. The motion of SCG was observed using a high-speed electronic camera in two configurations. In configuration 1, the camera recorded images of SCG in the welding gap along the direction of detonation (Figure 2a). Configuration 2 recorded images in the direction shown by the red arrow (Figure 2b). In order to have an unobstructed view of the motion of SCG across the entire width of the base plate, it was made from acrylic glass.

In the resulting images, the length of the SCG region was measured. Then, these measurements were compared with the results of the calculation. Then, the SCG temperature was measured through optical pyrometry.

### Initial Materials and Experimental Methods

Commercially available plates of 12Cr18Ni10Ti (AISI 321), AlMg6, and acrylic glass were used in the experiments. The initial plates had the dimensions of 3(4) × 200 × 300 mm.

**Figure 3:**
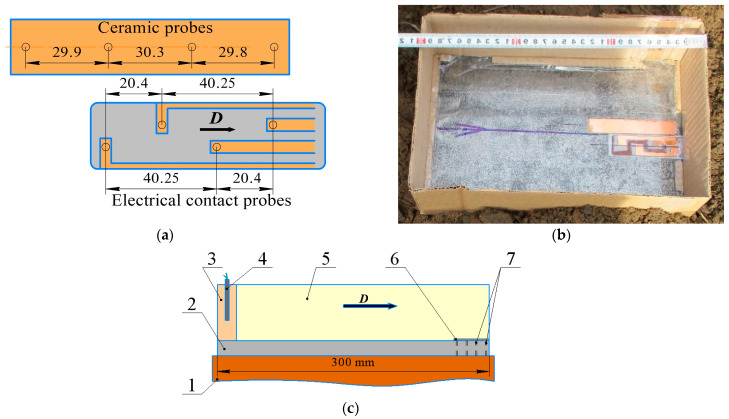
The measurement of the denotation velocity: (**a**) scheme of arrangement of probes; (**b**) photograph the steel plate with probes; (**c**) scheme of the assembly: 1—sand, 2—steel plate, 3—primary charge, 4—detonator, 5—secondary charge, 6—electrical contact probes, 7—ceramic probes.

Figure 3a shows the scheme of the arrangement of the electrical contact probes and ceramic probes. The probes were placed onto the steel plate and fixed by adhesive tape at the end edge of the plate (Figure 3b). The scheme of the assembly before EW is shown in Figure 3c. In order to capture the voltage signals from the probes a Tectronix DPO3054 oscilloscope (Tektronix (China) Co., Ltd., Shanghai, China) with clock frequency of 2.5 GHz was used. The time interval needed for the shock wave to pass the distance between two probes was measured by an electronic counter with clock frequency of 500 MHz. The denotation velocity was calculated as a ratio of distance to time.

**Figure 4:**
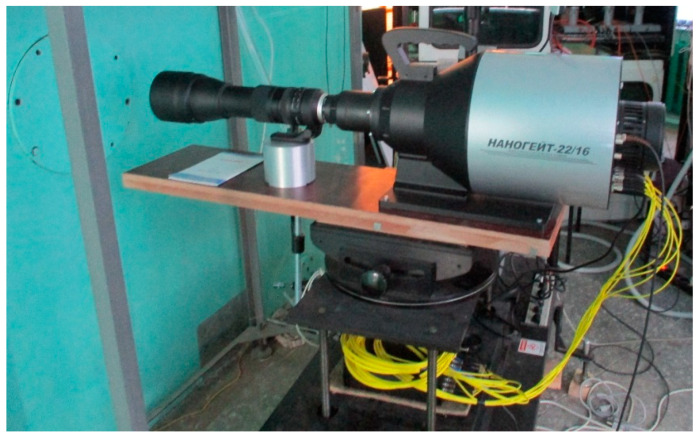
The photograph of NANOGATE-22/16 high-speed electronic camera.

The NANOGATE-22/16 high-speed electronic camera (Nanoskan, Moscow, Russia) (Figure 4) allowed us to record a series of images of rapid processes in visible spectrum. This camera is an 8-channel system that consists of an entrance lens and a catadioptric-lens unit for dividing an image into eight channels. As a result, 16 recorded images appear on a computer monitor. The electronic camera allowed us to set the time between frames from 2 ns to 100 μs. In configuration 1, the time between frames is 3.125 μs, and in configuration 2 is 7.300 μs. The image resolution is 2128 × 2112 px.

**Figure 5:**
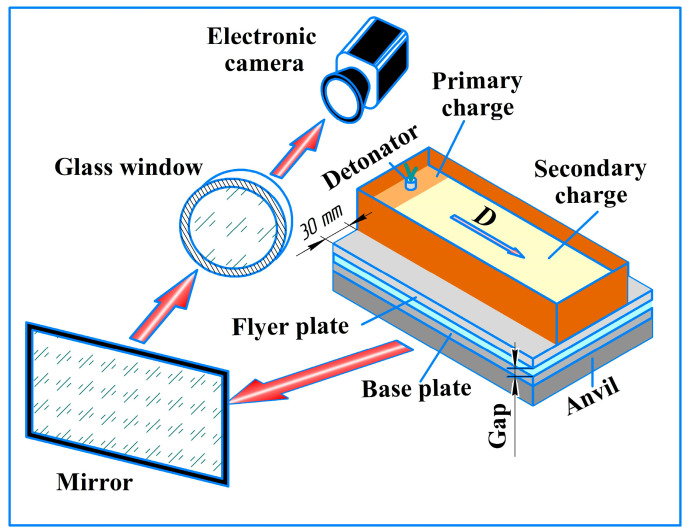
Schematic diagram of configuration 1.

**Figure 6:**
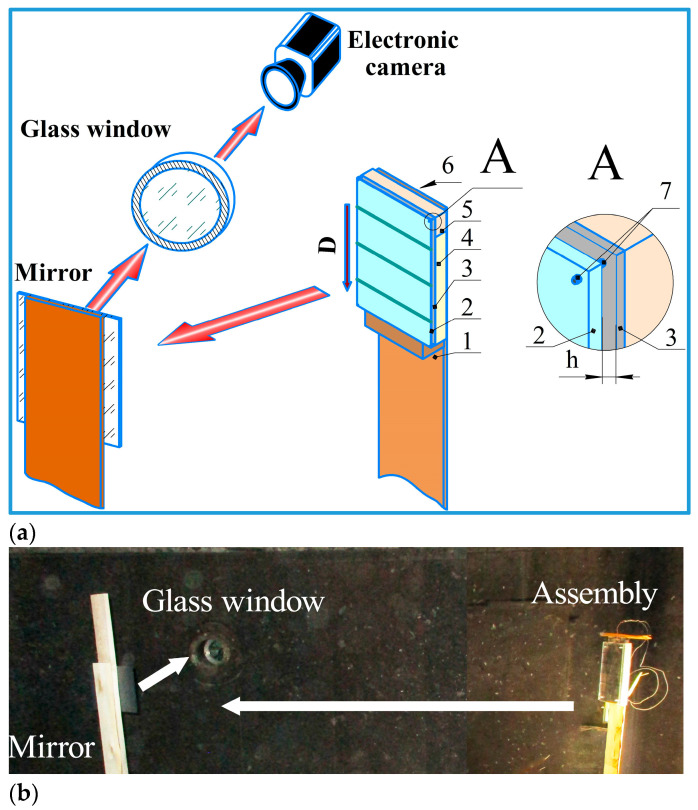
Configuration 2 for high-speed photography of SCG: (**a**) schematic diagram: 1—post; 2—base plate (acrylic glass); 3—flyer plate; 4—secondary charge; 5—primary charge; 6—detonator; 7—screw, (**b**) photograph of configuration 2.

This camera was used in two experimental configurations. Configuration 1 is shown in Figure 4. The explosive charge consisted of a primary charge and a secondary charge. The primary charge is ammonite and the secondary charge is an ANFO 96:4 mixture of microporous ammonium nitrate with diesel oil (d = 780 kg/m^3^). To prevent the detonation products from blocking the view of the welding gap during the process of photography, the width of the explosive was made narrower than the width of the flyer plate. The direction of the photography process in both configurations is shown by the red arrow (Figure 5 and Figure 6).

In configuration 2 (Figure 6a,b), a 4-mm-thick acrylic glass base plate the high-speed photography was performed on the side of the to observe the motion of SCG. The base plate had the lines painted at the 50 mm intervals to evaluate the velocity of SCG during EW.

**Figure 7:**
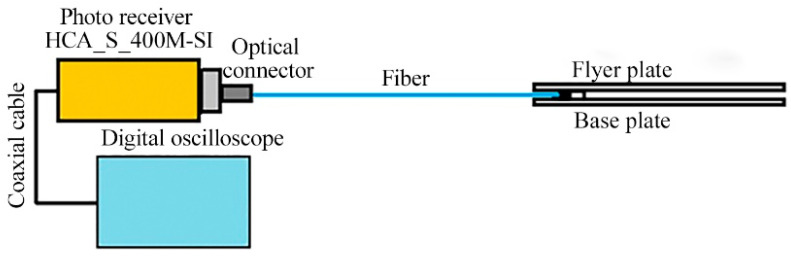
Schematic diagram of measurement of the SCG temperature.

Optical pyrometry was used to measure the SCG temperature in the welding gap according to the schematic diagram (Figure 7). Monitoring and recording of the radiation emitted by SCG was conducted using silicon photodiodes (with a photosensitive area of 0.8 mm^2^) and a FEMTO fast response amplifier (FEMTO, Berlin, Germany) with a frequency of 400 MHz. Also, the quartz light fiber and interference light filter with a width of 40 nm (wavelengths are 750 nm, 800 nm, 900 nm) were used to measure the SCG temperature.

**Figure 8:**
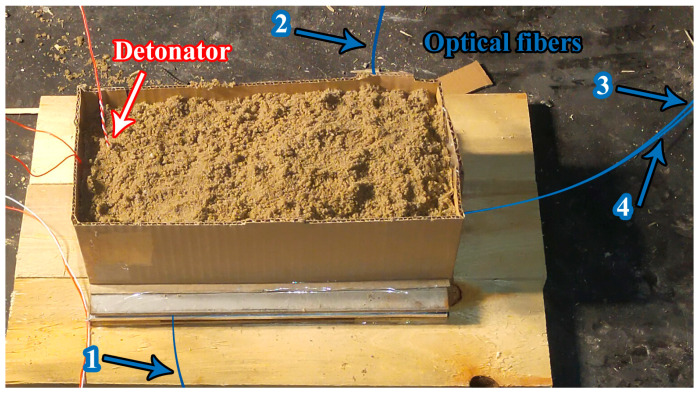
Photography of the scheme of location optical fibers for measurement of the SCG temperature (the numbers indicate optical fibers).

The scheme of the location of the optical fibers in the welding gap is shown in Figure 8. The optical fibers were attached by means of a glue to the base plate. Then, the flyer plate was placed and the explosive charge was extended over the flyer plate as in configuration 1. A layer of sand was placed on the explosive charge.

**Figure 9:**
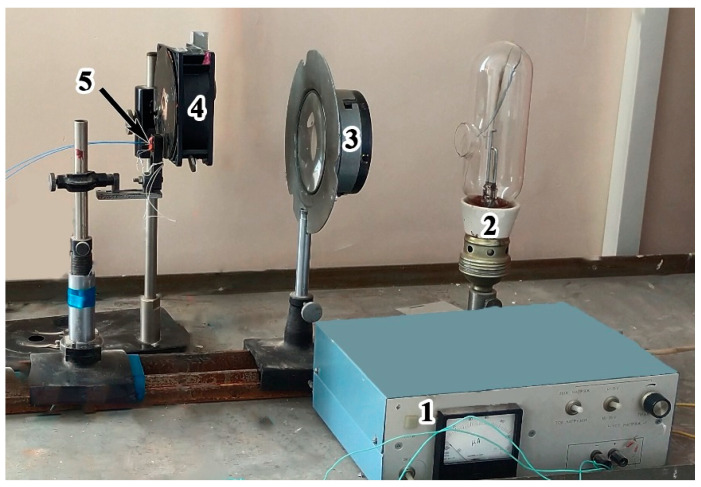
Photograph of the scheme of calibration of silicon photodiodes: 1—current source; 2—tungsten filament lamp; 3—collecting lens; 4—chopper; 5—optic fiber holder.

The silicon photodiodes were calibrated as follows: a tungsten filament lamp 2 was connected to a current source 1, turned on, and emitted radiance (Figure 9). The radiance intensity of the lamp was adjusted by changing the magnitude of the current. The radiance temperature at certain current values was determined using a precise pyrometer. As a result, the radiance temperature–current relation was obtained in the range of 1200–2400 K. Then, instead of a precise pyrometer, the optic fibers 5 and the silicon photodiodes were connected. Further, changing the current recorded the signal level of the optic fiber, thus obtaining a calibration curve. Thus, when the experiment (Figure 8) was conducted, the signal level was converted into the temperature. In the range of 2000–15,000 K the relative error is less than 10%.

This calibration scheme uses a chopper 3. The chopper is a rotating disk with a slot. Light passing through it hits the lens in pulses, and the digital oscilloscope, which registers the signal from the photodiode standing at the end of the light fiber during calibration is in the accumulation mode and forms an average signal.

The EW parameters were calculated from the equations taken from [31]. The equation for the flyer plate velocity is given as follows:

(1) $$\vartheta_{0}=1.2D\cdot \frac{\sqrt{1+\frac{32}{27}\cdot r}-1}{\sqrt{1+\frac{32}{27}\cdot r}+1},$$

where D is the detonation velocity and m/s; r is the ratio of the explosive mass to the flyer plate mass:

(2) $$r=\frac{m_{e}}{m_{pl}},$$

where *m_e_* is the explosive mass, kg; *m_pl_* is the flyer plate mass, kg.

Explosive welding parameters and dimensions of the initial materials are given in Table 1.

To calculate the thermodynamic parameters of a SCG, we used the shock adiabatic equations given in [32]:

(3) $$\frac{p_{1}}{p_{0}}=\frac{6-\frac{V_{1}}{V_{0}}}{\frac{8V_{1}}{V_{0}}-1}$$

(4) $$\frac{\rho_{0}}{\rho_{1}}=\frac{V_{1}}{V_{0}}=\frac{\frac{p_{1}}{p_{0}}+6}{\frac{8p_{1}}{p_{0}}+1}$$

(5) $$\frac{T_{1}}{T_{0}}=\frac{p_{1}V_{1}}{p_{0}V_{0}}$$

where P_1_ is the pressure in the SCG region, Pa; P_0_ is the atmospheric pressure (101,325 Pa); *V*_0_ is the specific volume of gas before compression, ($V_{0}=\frac{1}{\rho_{0}}$); *V*_1_ is the specific volume of gas after compression, ($V_{1}=\frac{1}{\rho_{1}}\;$);$\;\rho_{0}$ is the initial density of gas (1.3 kg/m^3^); $\rho_{1}$ is the density of gas after compression, kg/m^3^; T_1_ is the temperature of the SCG, K; T_0_ is initial temperature of gas (300 K).

It is more convenient to use the equation [32]:

(6) $$\frac{7}{5}M^{2}=\frac{\frac{p_{1}}{p_{0}}-1}{1-\frac{V_{1}}{V_{0}}}$$

(7) M=D/c

where M is the Mach number; D—the detonation velocity, m/s; c—the speed of sound in gas (331 m/s).

To calculate the pressure in the SCG region, Equation (4) was substituted into Equation (6). As a result, Equation (8) was obtained:

(8) $$\frac{7}{5}M^{2}=\frac{\frac{p_{1}}{p_{0}}-1}{1-\frac{\frac{p_{1}}{p_{0}}+6}{\frac{8p_{1}}{p_{0}}+1}},$$

The length of the SCG region was calculated from Equation (9) [19]:

(9) $$l=\frac{L\rho_{0}b}{\rho_{1}b+2\frac{L}{\vartheta_{c}}\sqrt{\frac{2\gamma }{\gamma +1}p_{1}\rho_{1}{\left(\frac{2}{\gamma +1}\right)}^{\frac{2}{\gamma -1}}}},$$

where *L* is the length of the plate, m; *γ* is the heat capacity ratio (1.4); *ϑ_c_* is the collision point velocity, m/s (it is equal to the detonation velocity).

## 3. Results

**Figure 10:**
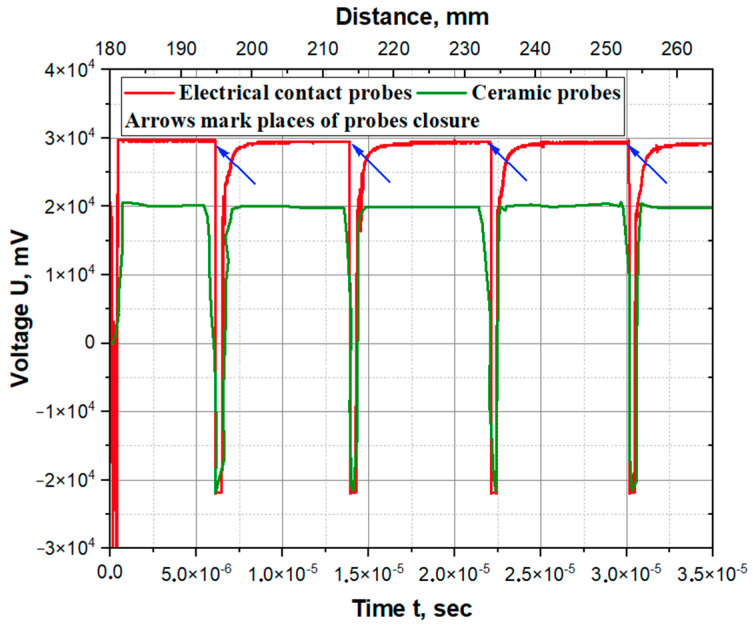
Oscillograms of detonation process.

Figure 10 shows the oscillograms of the detonation wave profiles as a function of time. The drop in signal amplitude recorded by the digital oscilloscope corresponds to the moments when probes close. The average detonation velocity measured using electrical contact probes and ceramic probes was 2470 and 2440 m/s, respectively. The oscillograms show that the detonation velocity is constant along the length of the explosive charge.

The results of calculating the thermodynamic parameters of SCG at the detonation velocity of 2500 m/s showed the following values: the pressure was 7.09 × 10^6^ Pa, the density was 9.62 kg/m^3^, the temperature was 2832 K.

**Figure 11:**
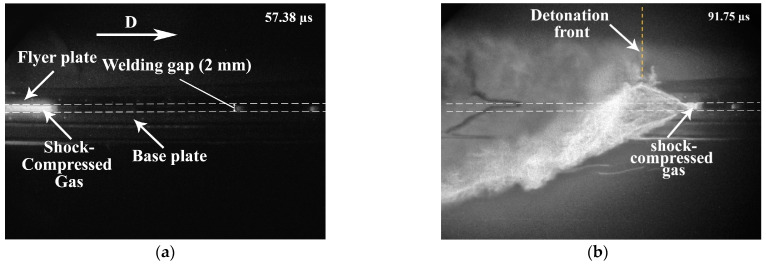
High-speed photography images (in configuration 1) over time: (**a**) 57.38 µs and (**b**) 91.75 µs.

Figure 11 shows the images of the SCG region in the welding gap captured in configuration 1. It can be seen that the head part of the SCG is ahead of the detonation front (Figure 11b). The head front of the SCG region had a velocity of 3000 ± 100 m/s.

**Figure 12:**
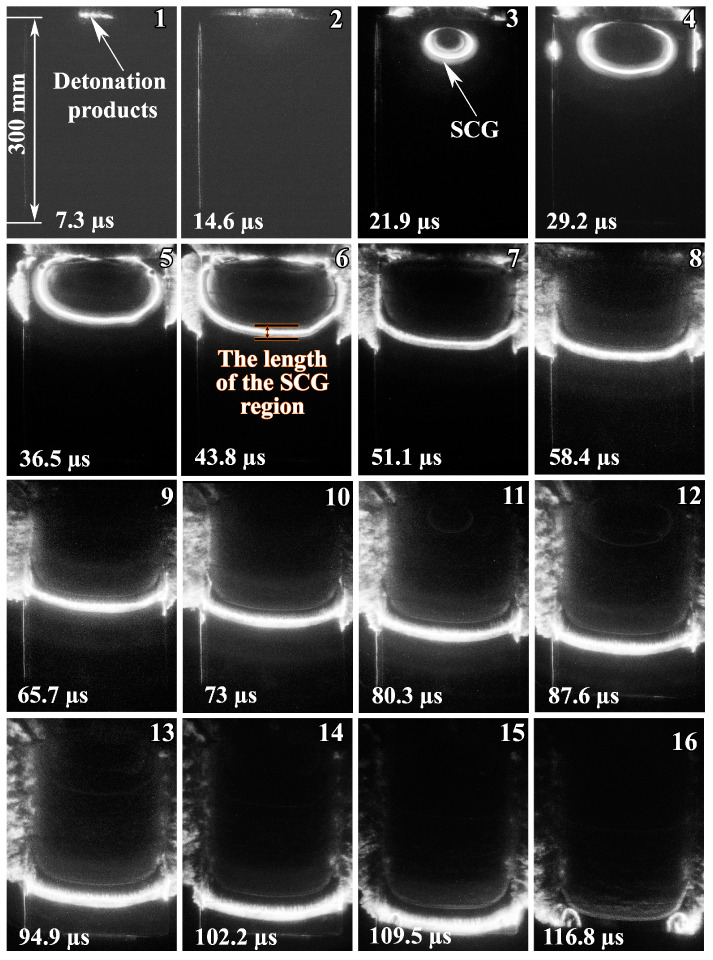
High-speed photography images of SCG region (in configuration 2) over time.

Figure 12 shows high-speed photography images of the SCG region captured in configuration 2. The first two images show glowing detonation products (Images 1 and 2). At this point in time, the flyer plate is still in the initial position. The elliptical SCG region around the initiation point was formed after collision (Image 3). As the contact point moved, the size of the elliptical SCG region increased (Images 4 and 5). As can be seen in Images 6–11, the SCG region flows out to the sides and takes on a linear shape. The velocity of the SCG region in the center was higher than the velocity of the peripheries. Then, these velocities become equal (Images 12–15). Image 16 shows the moment when the SCG region escapes into the atmosphere.

**Figure 13:**
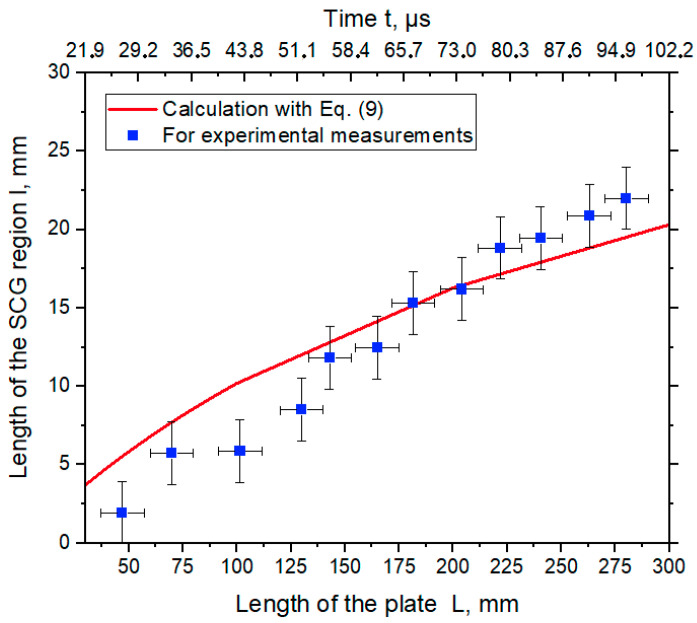
The length of the SCG region vs. length of the plate vs. time.

Figure 13 shows a plot of the change in the SCG region length calculated from Equation (9) and measured from photography images along the length of the plates.

The results of the calculation according to Equation (9) showed that the length of the SCG region increased as the collision point moved (Figure 13, red curve), and at a distance of 300 mm it was about 20 mm. Measurement from the images showed that at a distance of about 275 mm, the length of the SCG region was about 22 mm.

**Figure 14:**
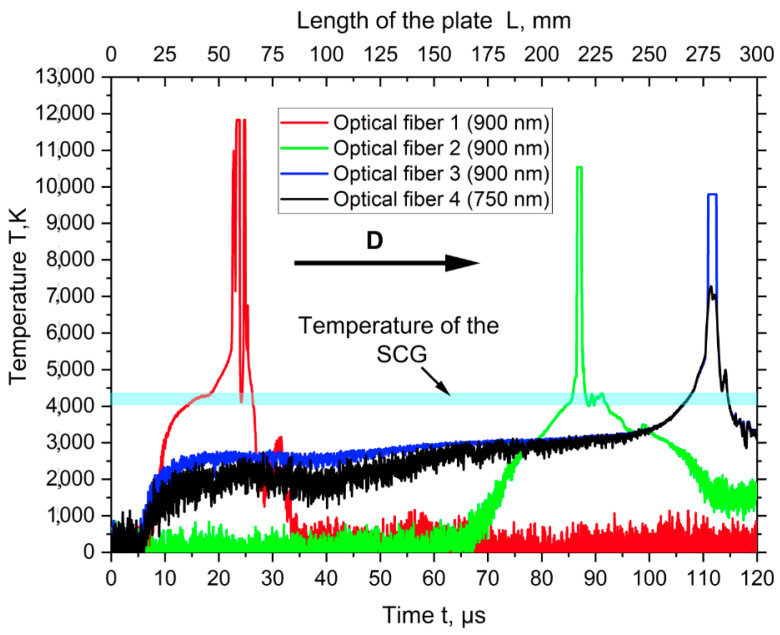
The oscillograms of the SCG region temperature.

**Figure 15:**
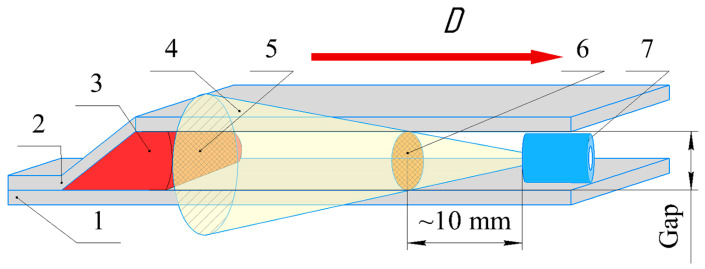
The scheme for determining the distance of maximum light collection from SCG region: 1—base plate; 2—flyer plate; 3—SCG region; 4—acceptance cone; 5—first field of view of the optical fibers; 6—second field; 7—optical fiber.

Figure 14 shows the oscillograms from the digital oscilloscope. Using the developed scheme for determining the location of maximum light collection (Figure 15), it was determined that the SCG temperature was 4100–4400 K (blue line). The peaks on the oscillograms are explained by the shock wave hitting the optical fiber.

Figure 15 shows the scheme for determining the distance of maximum light collection from the SCG region. Initially, the base area of the acceptance cone was larger than the welding gap area. Therefore, the field of view of the optical fibers included both the SCG region and the surfaces of the flyer and base plates. As the SCG region approached the optical fibers, the acceptance cone narrowed and the maximum light collection from the SCG region occurred approximately 10 mm from the edge of the optical fiber. Therefore, it can be assumed that at this distance, the SCG temperature was recorded close to the correct temperature.

## 4. Discussion

The calculation of the SCG parameters showed that the pressure in the SCG region is six times higher than the initial one, and the density is about nine times higher than the initial one. Thus, in the EW process there was a high compression of the SCG region, which increased the temperature in this region up to 2832 K. However, such temperatures cannot affect the surfaces of the plates due to the short length of the SCG region and the short time of exposure of SCG to the surfaces.

Based on the analysis of previous publications, it can be stated that the present work is the first to record in detail the motion process of the SCG region in the welding gap. High-speed photography according to configuration 1 confirmed the presence of the SCG region ahead of the collision point. The bright glow of this region indicates that the SCG region had a high temperature, which was confirmed by optical pyrometry measurements (Figure 14).

The images in configuration 2 (Figure 12) show that the SCG region had an elliptical shape due to the collision of the flyer plate with the base plate. When the SCG region reached the right and left edges of the plates, it began to flow out of the welding gap. After 94.9 μs (Figure 12, images 13–15), the SCG region had the stationary parameters, indicating a state of equilibrium between the masses of the compressing and outflowing gases.

Figure 12 shows two plots demonstrating how the length of the SCG region changes according to the calculation and the high-speed photography according to configuration 2. It can be seen that the results are identical: the length of the SCG region gradually increases as the EW process proceeds. It allowed use to use this calculation for the determination of the EW parameters of large plates, because as the length of the SCG region increases, the exposure time of the SCG region to the welded plates also increases. This phenomenon leads to the melting of the plate surface and the formation of various inhomogeneities at the weld interface and worsening of the mechanical properties of the weld joint.

The temperature of the SCG region was measured using the optical pyrometry. The main advantage of optical pyrometry is the possibility of remote temperature measurement without direct contact of the measuring device with the object. The maximum calculated temperature of the SCG was 2832 K, while the results of optical pyrometry showed a temperature in the range of 4100 to 4400 K. There seems to be only one explanation for this, namely, the availability of small metal particles into the SCG region. The SCG region has supersonic velocity and high density, which break these particles off the welded surfaces, carrying them with it. As a result of heat exchange between the hot particles and the SCG region, the particle is cooled and the SCG region is heated. These processes are not taken into account in the calculation due to complexity, so the calculated temperature is almost two times lower than the experimental one. In fact, the calculation gives the temperature value only from shock compression of the gas.

## 5. Conclusions

This paper demonstrates the potential for using the complex method of the determination of the SCG parameters. This method includes determination of the detonation velocity of a flat ANFO charge, high-speed photography of SCG region, and measurement of the SCG temperature using optical pyrometry. Simultaneous using the electrical contact and ceramic probes with the oscilloscope has made it possible to determine the detonation velocity with high accuracy. Based on a detonation velocity of 2500 m/s, the calculated SCG temperature was 2832 K, while the temperature was measured by optical pyrometry was in the range of 4100–4400 K. Presumably, this is due to the fact that small metal particles that broke off from the welded surfaces transferred their heat to the SCG region. The calculation cannot account for the heat transfer to the SCG region from metal particles. Two configurations for high-speed photography of the SCG region were developed and successfully applied. First, the images of the motion of SCG region across the entire width of the welded plates were obtained by using the acrylic plate. The study’s findings confirmed that the SCG region with high temperature is formed and moves at supersonic speed in the welding gap. This study yielded new data about the formation process and shape of the SCG region. As a result, these findings permit the more accurate selection of explosive welding modes.

## Figures and Tables

**Table 1:** Explosive welding parameters and dimensions of the initial materials.

Configuration	Base Plate (Dimensions, m)	Flyer Plate (Dimensions, m)	Detonation Velocity, m/s	Welding Gap, m	Flyer Plate Velocity, m/s	Explosive Ratio
Configuration 1	AlMg6 (0.004 × 0.2 × 0.3)	12Cr18Ni10Ti (0.004 × 0.2 × 0.3)	2500	0.002	526	0.9
Configuration 2	Acrylic glass (0.004 × 0.2 × 0.3)	12Cr18Ni10Ti (0.004 × 0.2 × 0.3)	2500	0.002	526	0.9

## Data Availability

Data are contained within the article.
